# Computer vision syndrome and its associated factors in health science students from a university in Peru

**DOI:** 10.3389/fpubh.2025.1485515

**Published:** 2025-04-28

**Authors:** Rocío Lopez-Choquegonza, Cesar Copaja-Corzo, Javier Flores-Cohaila

**Affiliations:** ^1^Facultad de Ciencias de la Salud, Universidad Privada de Tacna, Tacna, Peru; ^2^Unidad de investigación para la generación y síntesis de evidencias en salud, Universidad San Ignacio de Loyola, Lima, Peru; ^3^Servicio de Infectología, Hospital Nacional Edgardo Rebagliati Martins, EsSalud, Lima, Peru; ^4^Hospital Nacional Victor Larco Herrera, Lima, Peru

**Keywords:** computer vision syndrome, electronic devices, nomophobia, Peru, smartphone

## Abstract

The objective was to estimate the prevalence and identify the factors associated with Computer Vision Syndrome (CVS) among health science students at a university in Peru, we conducted a cross-sectional analytical study in Tacna, Peru, from October 12 to December 15, 2023. Participants were assessed through physical surveys. We evaluated 502 health science students; 59.2% were female, and the median age was 21 years (range 19 to 23 years). 78.1% had CVS, and 52% had moderate nomophobia. In the adjusted model, monthly family income of 2000 to 5,000 soles (PR: 0.81; 95% CI: 0.73 to 0.91) and over 5,000 soles (PR: 0.84; 95% CI: 0.74 to 0.95) was associated with a lower prevalence of CVS compared to families with income less than 2000 soles. Additionally, the prevalence of CVS was higher among students with moderate nomophobia (PR: 1.91; 95% CI: 1.24–3.16) and severe nomophobia (PR: 2.07; 95% CI: 1.31–3.48) compared to those with no symptoms or only mild symptoms of nomophobia. The prevalence of CVS is high among health science students, especially in medical students. As the severity of nomophobia increases, so does the prevalence of CVS, and higher family income is associated with lower CVS.

## Introduction

Computer Vision Syndrome (CVS) is characterized by ocular symptoms such as burning, itching, foreign body sensation, tearing, excessive blinking, blurred or double vision, red and dry eyes, difficulty focusing on near tasks, light sensitivity, visual halos, and headache (1); It is estimated that the prevalence of CVS worldwide is 66% (2), with percentages ranging from 35.2 to 97.3% in adults (3), and from 12.1 to 94.8% in children (4), making it a common issue (5). While the prevalence is based only on approximate estimates, the frequency of CVS in potential risk subgroups, such as university students, remains unclear, especially with the increased use of computers, tablets, e-readers, and smartphones for educational, communicative, and recreational purposes. Additionally, the COVID-19 pandemic further escalated the use of technological devices, potentially contributing to the continued increase in the prevalence of CVS (6, 7).

CVS has been reported to impact the quality of life of those affected (8). Other research has suggested that CVS could be associated with macular integrity issues and foveal dysfunction (9). In daily life, CVS might be linked to reduced productivity, visual and musculoskeletal impairment (10), as well as disruptions in circadian rhythms, altered sleep patterns, and increased anxiety and depression (11, 12). While some potential consequences of CVS have been reported, it is not yet clear what other issues it may entail.

Some factors associated with CVS have been identified, for example in Saudi Arabia, a study evaluating 300 medical students identified that factors associated with a higher prevalence of CVS were being female and using electronic devices for more extended periods (13). Another study conducted in Saudi Arabia, involving 521 students, found that older age, female gender, refractive errors, and the use of digital devices for more than 6 h were the main factors associated with CVS among university students (14). Another study in Thailand, which assessed a population of 527 university students attending virtual classes, found that being female, having previous ocular symptoms, astigmatism, screen distance <20 cm, screen reflections, low screen brightness, inadequate sleep duration between classes, and longer screen time was associated with CVS (15). Although these studies included a considerable number of participants, they were conducted online, potentially excluding certain student populations due to accessibility and connectivity limitations. This circumstance, along with selection bias and the limited extrapolation of data to other populations, is a point to consider. Recently, the issue of CVS has been studied in Latin America. A study conducted in Colombia reported a prevalence of 41.07% (16), while another study in Peru found a prevalence of 93% (17). Although these results are concerning, both studies focused exclusively on medical students and had small sample sizes, which limits the generalizability of their findings. Additionally, among the reported studies, only one (14), used regression models to identify the strength of the association between variables; the rest only employed hypothesis testing, further limiting the clarity of their results.

It is evident that more studies are needed to provide results that are extrapolatable to other realities, as well as to evaluate other associated factors to increase knowledge in this field and to develop more precise interventions aimed at reducing this problem. Therefore, this study aims to identify the prevalence and factors associated with CVS among health science students at a university in Peru.

## Methods

### Design and context

An analytical cross-sectional study was conducted on health science students at the Private University of Tacna (UPT) from October 12 to December 15, 2023. The Faculty of Health Sciences (FACSA) includes the schools of medicine, dentistry, and medical technology. UPT is a non-profit private institution affiliated with the Peruvian Association of Medical Schools (ASPEFAM) and the Peruvian Association of Dental Schools (ASPEFO). Additionally, it is one of the two universities in Tacna, Peru (18).

### Population

We included students from health science disciplines (Medicine, Dentistry, and Medical Technology, including Physical Therapy and Rehabilitation, Clinical Laboratory, and Pathological Anatomy) who provided informed consent. We decided to include these students because they tend to have a higher academic and financial burden to support their studies, which is reflected in multiple problems, including excessive use of electronic devices and, more frequently, the development of CVS compared to other students (17–20). Those with incomplete surveys (more than 10 missing data points) or those who decided to withdraw from the study during the survey were excluded.

A *post hoc* power analysis was conducted using the one-sample proportion test (power oneprop) in Stata. Considering an expected proportion of 93% (Meneses et al.) (17), an observed proportion of 78.1%, a sample size of 502 participants, and a significance level of 0.05, a statistical power of 99% was obtained.

### Procedures

The data collection was carried out from November to December 2023. Entry into the classrooms was punctual at the agreed-upon time with the course instructor during class hours. Upon entry, Rocio Lopez (RL) introduced herself to the students as part of the research team, explained the research objective, provided details about voluntary participation, and explained the process of signing the informed consent if they chose to participate. She also gave the students guidelines on how to fill in each section of the data collection form.

During the survey, RL informed the students that if they had any questions while completing the form, they could raise their hand, and the researcher would approach them to address their concerns. Additionally, they were instructed to raise their hand after completing the form so RL could collect it. After this, they were told they could begin, and the average time it took for students to complete the surveys was 15 min. After all participants in the classroom had finished filling out the forms and collected all the surveys, they were thanked for their participation.

After collecting the information in physical form, the data was transferred to an Excel data collection form. Data entry was done twice by different researchers [Cesar Copaja (CC) and RL]. After completing this process, both datasets were reviewed to identify inconsistencies in the data recording. If any data differed, the physical form was consulted, and the error was corrected.

### Instrument and variables

The data collection form was anonymous, and no information that could identify the participant was requested. The form consisted of three sections: First, sociodemographic characteristics (08 questions); second, use of technological devices (06 questions); and third, CVS (16 items) and nomophobia (20 questions). The complete data collection form can be found in Appendix A1.

The dependent variable was CVS. For its determination, we used the Computer Vision Syndrome Questionnaire (CVS-Q). It is a self-administered questionnaire consisting of 16 items, designed initially by Seguí et al. in 2015 in English for administrative workers (19). Our study used the validated version for Peruvian healthcare professionals (20), which reported adequate internal consistency (Cronbach’s Alpha 0.939). For interpreting the CVS-Q questionnaire, the product obtained from the intensity and frequency allows us to calculate the severity of each symptom. If the sum found from this calculation is greater than or equal to 06 points, it can be affirmed that the person has symptoms of CVS (19).

To assess nomophobia, defined as the fear of not having contact with a mobile phone (21), we used the Nomophobia Questionnaire (NMP-Q), which evaluates its severity. This is a self-report questionnaire consisting of 20 items with a 7-point Likert scale, ranging from 1 (“strongly disagree”) to 7 (“strongly agree”). The NMP-Q was originally developed in English by Yildirim and Correia (22), In our study, we used the Spanish version validated in a population aged 13 to 19 years (23), also utilized in a similar study with university students in Peru aged 17 to 34 years, reporting overall good internal consistency (Cronbach’s Alpha 0.964) (24). For interpretation, the following criteria were considered: a total score of 20 indicates the absence of nomophobia; a score greater than 20 and less than 60 indicates a mild level of nomophobia; a total score greater than or equal to 60 and less than 100 indicates a moderate level of nomophobia; and a total score greater than or equal to 100 indicates severe nomophobia (22).

### Statistics

The analyses were performed using the statistical software RStudio. To describe the population and its characteristics, we employed frequencies, percentages, measures of central tendency, and dispersion.

We utilized Poisson regression models with robust variance to address our research question. Given that this is an exploratory study, we decided to perform univariable selection to determine the variables that would enter the adjusted model (25, 26). For this, a crude analysis was performed between each variable and the outcome (CVS), and statistically significant variables (*p* < 0.05) were included in the multivariate model. We obtained adjusted prevalence ratios (aPR) and their respective 95% confidence intervals (CI 95%) in the multivariate analysis.

## Results

**Figure 1:**
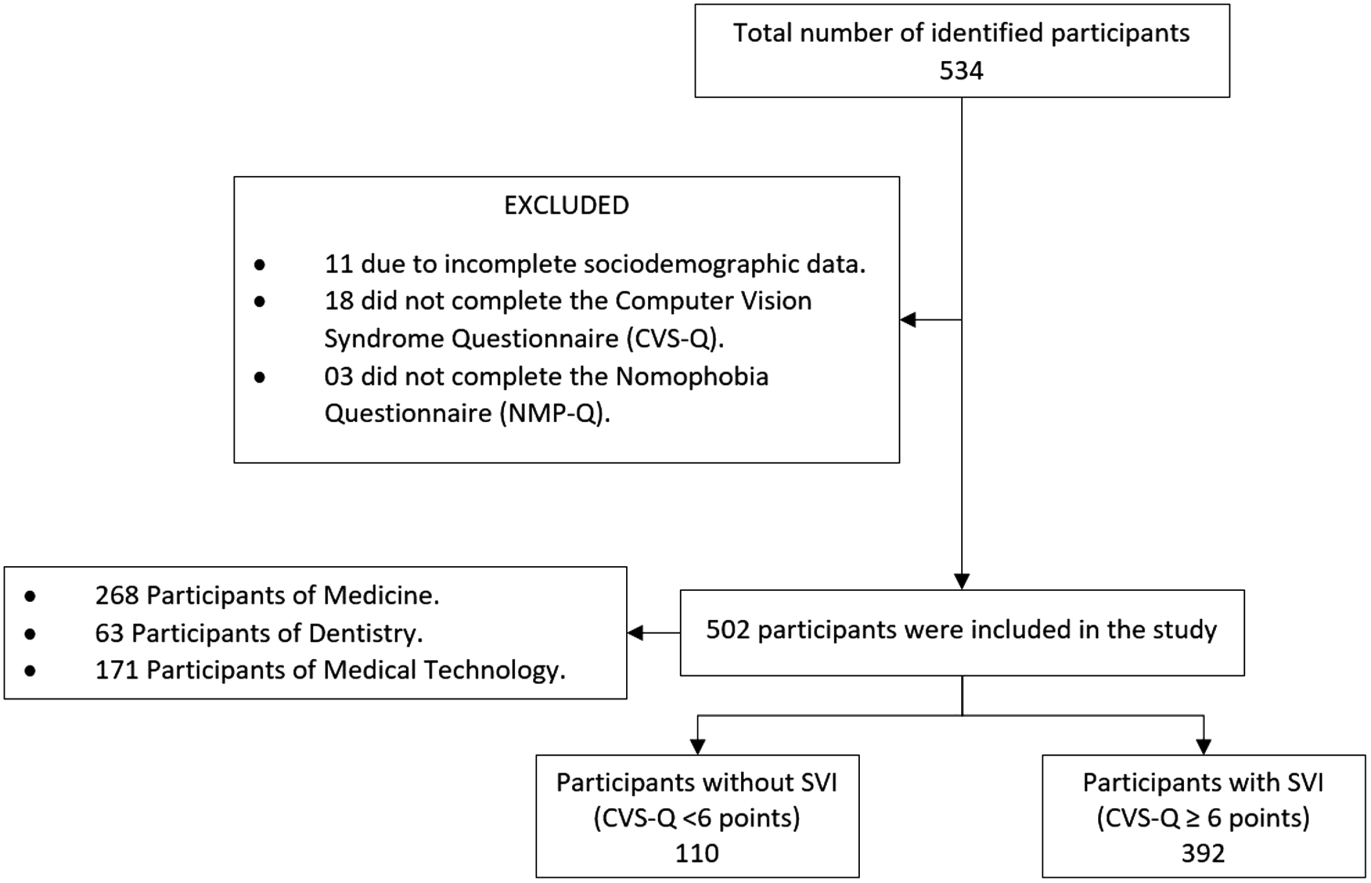
Selection flowchart. CVS-Q, Computer Vision Syndrome Questionnaire; CVS, Computer Vision Syndrome; NMP-Q, Nomophobia Questionnaire.

A total of 534 health science students were surveyed. Out of the total, 32 surveys were excluded as they did not meet the inclusion criteria, and ultimately, the surveys of 502 students were analyzed (Figure 1).

### Population characteristics

The median age was 21 years (range 19 to 23 years), and 59.2% were women. Most students (53.4%) were studying medicine, and 65.7% were in the 1st to 3rd year of study.

Women had a higher prevalence of CVS (80.1%) compared to men (75.1%). Regarding the use of technological devices, 30.5% reported using their cell phones between 4 and 6 h per day. Concerning the NMP-Q scale, 52% had moderate nomophobia, and 7.4% had severe nomophobia. On the other hand, 78.1% experienced computer vision syndrome (6 points or more; Table 1).

**Table 1:** Population Characteristics (*n* = 502).

Characteristics	n (%)
Sex	
Female	297 (59.2)
Male	205 (40.8)
Age	
≤ 22 years old	347 (69.1)
≥ 23 years old	155 (30.9)
Siblings	
No siblings	66 (13.1)
With siblings (≥ 1 sibling)	436 (86.9)
Health science disciplines	
Medicine	268 (53.4)
Dentistry	63 (12.5)
Medical Technology	171 (34.1)
Academic year	
≤ 3rd year	330 (65.7)
≥ 4th year	172 (34.3)
Monthly family income	
< 2000	144 (28.7)
2000–5,000	230 (45.8)
> 5,000	128 (25.5)
Father’s highest level of education	
No education or only school education	94 (18.7)
Only with technical studies	134 (26.7)
Only with university studies	274 (54.6)
Mother’s highest level of education	
No education or only school education	110 (21.9)
Only with technical studies	122 (24.3)
Only with university studies	270 (53.8)
Continuous computer, laptop, or tablet usage time	
Less than 2 h	34 (6.8)
2–4 h	176 (35.1)
5–6 h	183 (36.5)
More than 6 h	109 (21.6)
Continuous smartphone/cell phone usage time	
Less than 2 h	79 (15.7)
2–4 h	136 (27.1)
5–6 h	153 (30.5)
More than 6 h	134 (26.7)
Most used technological device	
Laptop – computer	144 (28.7)
Tablet	46 (9.2)
Cellphone	312 (62.1)
Most used technological devices	
Laptop and Tablet	45 (9.0)
Laptop and cell phone	373 (74.3)
Tablet and cell phone	84 (16.7)
Internet access	
No	6 (1.2)
Yes	496 (98.8)
Type of internet access	
Only has Wi-Fi at home	63 (12.5)
Has Wi-Fi and data plan	427 (85.1)
Only has data plan at home	12 (2.4)
Computer Vision Syndrome (CVS)	
Without SVI (< 6)	110 (21.9)
With SVI (≥ 6)	392 (78.1)
Nomophobia NMP-Q	
Absence (≤ 20)	9 (1.8)
Mild (≤ 59)	195 (38.8)
Moderate (≤ 99)	261 (52.0)
Severe (≥100)	37 (7.4)

CVS-Q, Computer Vision Syndrome Questionnaire; CVS, Computer Vision Syndrome; NMP-Q, Nomophobia Questionnaire.

### CVS symptoms and nomophobia score

Participants with more frequent CVS symptoms had higher scores on the nomophobia questionnaire (NMP-Q). For example, those who experienced double vision “often or always” had an average NMP-Q score of 78.8 ± 25.1, while those who never experienced it scored 60.7 ± 23.5. Similarly, participants with frequent eye pain had a score of 76.9 ± 21.1, compared to 59.1 ± 24.6 in those who did not report this symptom (Table 2).

**Table 2:** CVS symptoms and nomophobia scores in health science students.

CVS-Q questionnaire	NMP-Q questionnaire (Mean ± SD)
1. In your eyes, when looking at a technological device, do you experience burning sensation?	
Never	56.2 ± 23.2
Occasionally	66.5 ± 23.7
Often or always	74.4 ± 21.1
2. In your eyes, when looking at a technological device, do you experience itching?	
Never	59.8 ± 23.8
Occasionally	66.6 ± 23.5
Often or always	74.4 ± 23.2
3. In your eyes, when looking at a technological device, do you feel as if you have something inside your eye?	
Never	61.0 ± 23.8
Occasionally	67.1 ± 23.1
Often or always	74.2 ± 25.1
4. In your eyes, when looking at a technological device, do you experience excessive tearing?	
Never	59.6 ± 22.0
Occasionally	65.6 ± 23.6
Often or always	73.1 ± 27.4
5. In your eyes, when looking at a technological device, do you experience excessive blinking?	
Never	59.0 ± 22.8
Occasionally	68.5 ± 23.8
Often or always	74.6 ± 24.1
6. In your eyes, when looking at a technological device, do you notice redness?	
Never	58.2 ± 22.0
Occasionally	68.8 ± 23.9
Often or always	71.8 ± 25.9
7. In your eyes, when looking at a technological device, do you experience eye pain?	
Never	59.1 ± 24.6
Occasionally	65.6 ± 22.9
Often or always	76.9 ± 21.1
8. In your eyes, when looking at a technological device, do your eyelids feel heavy or swollen?	
Never	58.9 ± 23.5
Occasionally	68.3 ± 23.1
Often or always	73.1 ± 24.4
9. In your eyes, when looking at a technological device, do you experience dryness?	
Never	60.3 ± 24.6
Occasionally	68.2 ± 22.2
Often or always	71.9 ± 24.0
10. In your eyes, when looking at a technological device, do you experience blurred vision?	
Never	59.9 ± 24.3
Occasionally	67.8 ± 23.4
Often or always	71.7 ± 21.3
11. In your eyes, when looking at a technological device, do you experience double vision?	
Never	60.7 ± 23.5
Occasionally	71.7 ± 22.0
Often or always	78.8 ± 25.1
12. In your eyes, when looking at a technological device, do you have difficulty seeing up close?	
Never	63.1 ± 23.9
Occasionally	66.3 ± 23.8
Often or always	68.9 ± 24.5
13. In your eyes, when looking at a technological device, do you experience increased sensitivity to light?	
Never	57.3 ± 23.7
Occasionally	66.1 ± 23.1
Often or always	74.1 ± 23.0
14. In your eyes, when looking at a technological device, do you see halos or circles around objects?	
Never	62.2 ± 23.5
Occasionally	67.0 ± 23.7
Often or always	73.3 ± 25.3
15. In your eyes, when looking at a technological device, do you feel like your vision has worsened?	
Never	60.2 ± 23.7
Occasionally	68.2 ± 22.9
Often or always	76.2 ± 23.6
16. When looking at a technological device, do you experience headaches?	
Never	60.3 ± 23.5
Occasionally	63.6 ± 23.1
Often or always	75.8 ± 24.0

### Factors associated with CVS

In the adjusted Poisson regression model with robust variance, we identified that a monthly family income of 2000 to 5,000 soles (aPR: 0.81; 95% CI: 0.73 to 0.91) and more than 5,000 soles (aPR: 0.84; 95% CI: 0.74 to 0.95) was associated with a lower prevalence of CVS compared to those earning less than 2000 soles per month. Additionally, the prevalence of CVS was higher in students with moderate nomophobia symptoms (PR: 1.91; 95% CI: 1.24 to 3.16), and severe nomophobia (PR: 2.07; 95% CI: 1.31 to 3.48) in comparison to those without symptoms or with mild symptoms of nomophobia (Table 3).

**Table 3:** Characteristics associated with computer vision syndrome (*n* = 502).

Characteristics	Without CVS (*n* = 110)	With CVS (*n* = 392)	Crude PR (95% CI)	Adjusted PR (95% CI)
Sex				
Female	59 (19.9)	238 (80.1)	Ref	–
Male	51 (24.9)	154 (75.1)	0.94 (0.86 to 1.03)	–
Age				
≤ 22 years old	73 (21.0)	274 (79.0)	Ref	–
≥ 23 years old	37 (23.9)	118 (76.1)	0.96 (0.87 to 1.07)	–
Siblings				
No siblings	17 (25.8)	49 (74.2)	Ref	–
With siblings (≥ 1 sibling)	93 (21.3)	343 (78.7)	1.06 (0.92 to 1.22)	–
Health science discipline				
Medicine	62 (23.1)	206 (76.9)	Ref	
Dentistry	21 (33.3)	42 (66.7)	0.87 (0.74 to 1.01)	–
Medical Technology	27 (15.8)	144 (84.2)	1.09 (0.99 to 1.21)	–
Academic year				
≤ 3rd year	72 (21.8)	258 (78.2)	Ref	–
≥ 4th year	38 (22.1)	134 (77.9)	1.00 (0.90 to 1.10)	–
Monthly family income				
< 2000	17 (11.8)	127 (88.2)	Ref	Ref
2000–5,000	61 (26.5)	169 (73.5)	0.83 (0.75 to 0.93)	0.81 (0.73 to 0.91)
> 5,000	32 (25.0)	96 (75.0)	0.85 (0.75 to 0.96)	0.84 (0.74 to 0.95)
Father’s highest level of education				
No education or only school education	16 (17.0)	78 (83.0)	Ref	–
Only with technical studies	31 (23.1)	103 (76.9)	0.93 (0.81 to 1.06)	–
Only with university studies	63 (23.0)	211 (77.0)	0.93 (0.82 to 1.05)	–
Mother’s highest level of education				
No education or only school education	22 (20.0)	88 (80.0)	Ref	–
Only with technical studies	21 (17.2)	101 (82.8)	1.03 (0.91 to 1.18)	–
Only with university studies	67 (24.8)	203 (75.2)	0.94 (0.84 to 1.06)	–
Continuous computer, laptop or tablet usage time				
Less than 2 h	9 (26.5)	25 (73.5)	Ref	–
2–4 h	45 (25.6)	131 (74.4)	1.01 (0.83 to 1.24)	–
5–6 h	36 (19.7)	147 (80.3)	1.09 (0.90 to 1.34)	–
Más de 6 h	20 (18.3)	89 (81.7)	1.11 (0.90 to 1.37)	–
Continuous smartphone/cell phone usage time				
Less than 2 h	23 (29.1)	56 (70.9)	Ref	–
2–4 h	32 (23.5)	104 (76.5)	1.08 (0.93 to 1.26)	–
5–6 h	31 (20.3)	122 (79.7)	1.12 (0.97 to 1.31)	–
Más de 6 h	24 (18.0)	110 (82.0)	1.16 (1.00 to 1.35)	–
Most used technological device				
Laptop – computer	33 (23.0)	111 (77.0)	Ref	–
Tablet	11 (24.0)	35 (76.0)	0.99 (0.82 to 1.18)	–
Cell phone	66 (21.2)	246 (78.8)	1.02 (0.92 to 1.14)	–
Most used technological devices				
Laptop and Tablet	14 (31.1)	31 (68.9)	Ref	–
Laptop and cell phone	81 (21.7)	292 (78.3)	1.14 (0.96 to 1.36)	–
Tablet and cell phone	15 (17.9)	69 (82.1)	1.19 (0.98 to 1.46)	–
Internet access				
No	1 (16.7)	5 (83.3)	Ref	–
Yes	109 (22.0)	387 (78.0)	0.94 (0.64 to 1.46)	–
Type of internet access				
Only has wifi at home	14 (22.2)	49 (77.8)	Ref	–
Has wifi and data plan	92 (21.5)	335 (78.5)	1.01 (0.88 to 1.16)	–
Only has data plan at home	4 (33.3)	8 (66.7)	1.86 (0.59 to 1.20)	–
Nomophobia				
Absence or Mild (≤ 59)	63 (30.9)	141 (69.1)	Ref	Ref
Moderate (≤ 99)	44 (16.9)	217 (83.1)	1.08 (1.03 to 1.13)	1.91 (1.24 to 3.16)
Severe (≥100)	3 (8.1)	34 (91.9)	1.13 (1.07 to 1.20)	2.07 (1.31 to 3.48)

CVS, Computer Vision Syndrome; NMP-Q, Nomophobia Questionnaire; Ref, Reference; CI, Confidence Interval; RP, Prevalence Ratio.

## Discussion

### Summary of findings

Here, we conducted a cross-sectional analytical study to investigate the prevalence of CVS, its association with nomophobia, and other related factors in 502 health sciences students from a Peruvian university. Our main findings were as follows: (1) The prevalence of CVS symptoms in this population was 78.1%; (2) There was an association between nomophobia severity and CVS, with the highest between severe nomophobia and CVS (PR: 2.07; 95% CI: 1.31 to 3.48), (3) A higher family income was associated with lower prevalence of CVS.

### Comparison with prior work

We found a prevalence of 78.1% of CVS. This was like a prior systematic review that reported a global prevalence of 66% (27). In health sciences education, a study conducted in Paraguay reported a prevalence of 82.5% (28), while another study conducted in Peru reported a prevalence of 58% (29). These findings highlight the importance of CVS. One major explanation, as described by Coronel (28), is the conversion of classes from a physical to a digital space after the COVID-19 pandemic, which led to CVS. Moreover, the widespread use of mobile phones and increased screen time may be associated with the high prevalence of CVS.

We identified an inverse association between family income and CVS, and a positive association between the severity of nomophobia and CVS. A study conducted in Ethiopia reported that participants with higher monthly incomes were 54.7% less likely to develop CVS compared to those with lower average monthly incomes (30). From the perspective of social determinants, higher incomes can influence other protective factors such as access to health education resources, healthcare awareness, and preventive measures, in addition to better equipment like light filters (2). This could explain our results.

We found an association between nomophobia and CVS, which has not been previously studied in the literature. The underlying mechanism behind this association could be attributed to the increased screen time and prolonged use of digital devices associated with nomophobia behavior. Individuals becoming more dependent on smartphones may engage in extended periods of close-range focusing, leading to CVS symptoms. This is of significant importance due to the high nomophobia reported prevalence worldwide among university students, reaching almost 60% for moderate and 20% for severe nomophobia (31). Therefore, in the new and evolving digital world, where future generations may be more accustomed to smartphones and other electronic devices, it is necessary to further study this association. Nomophobia has been linked to other outcomes such as anxiety, depression, low self-esteem, and lower academic performance (32–35). However, now it seems to also be associated with CVS.

### Implications

To our knowledge, this is the first study to address the association between CVS and nomophobia. Hence, this finding has several implications. For deans and faculty, the high prevalence of CVS represents a call to action for screening and early intervention strategies, integrating activities with no screens into the formal curriculum or digital wellness programs. For medical practitioners, the newly found association between nomophobia and CVS may require that when CVS is suspected, a screening of nomophobia be considered in companion to healthy digital habits and education regarding potential consequences of excessive digital device use. Researchers need to understand the association between nomophobia and CVS better, as this has not been previously explored, and consider the role of other social determinants as moderators.

## Strengths and limitations

This study has limitations that should be considered. Firstly, being a cross-sectional study, we cannot determine the temporality of the associations. Additionally, since the questionnaires were self-administered, social desirability bias might be present; however, participants were informed that the survey would be anonymous, which could reduce this risk. Additionally, we were unable to perform a comprehensive ophthalmologic examination, which would have provided a more in-depth analysis of the students’ eye health. Despite its limitations, this is, to the best of our knowledge, the first study to evaluate computer vision syndrome in health sciences students in Peru. We used rigorous inclusion criteria and an adequate statistical power to ensure the validity and inference of our results.

## Conclusion

In conclusion, this cross-sectional study among health sciences students in Peru found a high prevalence of CVS and a significant association between nomophobia severity and CVS, particularly between severe nomophobia and CVS. Additionally, higher family income was associated with a lower prevalence of CVS. These findings underscore the importance of addressing the growing issue of CVS and its potential link to nomophobia in the digital age. As smartphones and other electronic devices continue to rise, promoting healthy digital habits and raising awareness about the potential consequences of excessive digital device use on ocular health and overall well-being is crucial. It is crucial to develop new interventions aimed at reducing excessive smartphone use.

## Data Availability

The original contributions presented in the study are included in the article/Supplementary material, further inquiries can be directed to the corresponding author.
