# Supplementary material for: Computer vision syndrome and its associated factors in health science students from a university in Peru
Source: Front Public Health. 2025 Apr 28;13:1485515. doi: 10.3389/fpubh.2025.1485515 (PMC12066538; doi:10.3389/fpubh.2025.1485515)
Supplement: Supplementary file 1 [file Table_1.DOCX]

**QUESTIONNAIRE**

1. Gender ● Female (0) ● Male (1)
2. Age (What is your age in completed years) ● ______
3. How many siblings do you have? ● ______
4. To which professional school do you belong?

● School of Human Medicine (1)

● School of Dentistry (2)

● School of Medical Technology (3)

1. In which academic year are you enrolled?

● First year (1)

● Second year (2)

● Third year (3)

● Fourth year (4)

● Fifth year (5)

● Sixth year (6)

1. What is the average monthly income for your entire family?

● < 990 soles (1)

● 991-2000 soles (2)

● 2000-5000 soles (3)

● > 5000 soles (4)

1. What is the highest level of education attained by your father?

● No formal education (1)

● Only primary school (2)

● Only technical education (3)

● University education only (4)

1. What is the highest level of education attained by your mother?

● No formal education (1)

● Only primary school (2)

● Only technical education (3)

● University education only (4)

1. How much time per day do you use your computer or laptop without interruptions?

● Less than 2 hours (1)

● 2-4 hours (2)

● 4-6 hours (3)

● More than 6 hours (4)

1. How much time per day do you use your smartphone/cellphone without interruptions?

● Less than 2 hours (1)

● 2-4 hours (2)

● 4-6 hours (3)

● More than 6 hours (4)

1. Which technological device do you use the most during the day?

● Laptop – computer (1)

● Tablet (2)

● Smartphone (3)

1. Which technological devices do you use the most during the day?

● Laptop and Tablet (1)

● Laptop and smartphone (2)

● Tablet and smartphone (3)

1. Do you have access to the internet?

● I have no access at all (no Wi-Fi or data plan) (1)

● Yes, I have some form of internet access (2)

1. What type of internet access do you have?

● Only Wi-Fi at home (1)

● Have Wi-Fi and a data plan (2)

● Only have a data plan at home (3)

**CVS-Q QUESTIONNAIRE**

Indicate if you perceive any eye and visual symptoms during the use of technological devices (laptop/computer, tablet, smartphone). For each symptom, select the option that you consider correct. First, mark the frequency with which the symptom appears, considering the following:

● Never = On no occasion

● Occasionally = Sporadically or once a week

● Often or always = 2 or 3 times a week or almost every day

Second, mark the intensity with which you feel the discomfort:

● Remember: If you mark never in frequency, you should not mark anything in intensity.

**CUESTIONARIO CVS -Q**

Indique si percibe alguno de los síntomas oculares y visuales, a lo largo del tiempo de uso del dispositivo tecnológico (laptop/computadora, tablet, smartphone). Para cada síntoma, seleccione la opción que considere correcta.

En primer lugar, marque la frecuencia con que aparece el síntoma teniendo en cuenta lo siguiente:

- **Nunca** = En ninguna ocasión
- **Ocasionalmente** = De forma esporádica o una vez por semana
- **A menudo o siempre** = 2 o 3 veces por semana o casi todos los días

En segundo lugar, marque la intensidad con que la que siente la molestia:

- Recuerde: Si marca nunca en frecuencia, no debe marcar nada en intensidad

|  | **Frequency** | | | **Intensity** | |
| --- | --- | --- | --- | --- | --- |
| In the eyes when looking at the technological device, do you experience | Never | Occasionally | Often or always | Moderate | Intense |
| 1. Burning |  |  |  |  |  |
| 1. Itching |  |  |  |  |  |
| 1. Feeling of having something in the eye |  |  |  |  |  |
| 1. Tearing |  |  |  |  |  |
| 1. Excessive blinking |  |  |  |  |  |
| 1. Red eyes |  |  |  |  |  |
| 1. Eye pain |  |  |  |  |  |
| 1. Heavy or swollen eyelids |  |  |  |  |  |
| 1. Dry eyes |  |  |  |  |  |
| 1. Blurred vision |  |  |  |  |  |
| 1. Double vision |  |  |  |  |  |
| 1. Difficulty seeing up close |  |  |  |  |  |
| 1. Greater sensitivity to light |  |  |  |  |  |
| 1. Circles or lights around objects |  |  |  |  |  |
| 1. Feeling of seeing worse |  |  |  |  |  |
| 1. Headache |  |  |  |  |  |

**NMP-Q Questionnaire**

Please indicate how much you agree or disagree with each statement regarding your cellphone (smartphone). From 1 (totally disagree) to 7 (totally agree).

|  | **Very Disagree** | | | | **Very Agree** | | |
| --- | --- | --- | --- | --- | --- | --- | --- |
|  | 1 | 2 | 3 | 4 | 5 | 6 | 7 |
| 1. I would feel uncomfortable without constant access to information through my smartphone |  |  |  |  |  |  |  |
| 1. I would feel irritated if I could not search for information on my smartphone whenever I wanted |  |  |  |  |  |  |  |
| 1. I would be nervous if I could not get news (e.g., events, weather, etc.) on my smartphone |  |  |  |  |  |  |  |
| 1. I would be irritated if I could not use my smartphone and its capabilities when I wanted |  |  |  |  |  |  |  |
| 1. I would be scared to run out of battery on my smartphone. |  |  |  |  |  |  |  |
| 1. I would panic if I ran out of credit or exceeded my monthly data limit. |  |  |  |  |  |  |  |
| 1. If I had no data coverage or could not connect to Wi-Fi, I would constantly check if I have a signal or could find a Wi-Fi network. |  |  |  |  |  |  |  |
| 1. If I could not use my smartphone, I would be afraid of being stranded somewhere. |  |  |  |  |  |  |  |
| 1. If I were without checking my smartphone for a while, I would wish I could look at it. |  |  |  |  |  |  |  |
| 1. I would feel anxious if I could not communicate instantly with my family and friends. |  |  |  |  |  |  |  |
| 1. I would be worried because my family and friends could not contact me. |  |  |  |  |  |  |  |
| 1. I would be nervous because I could not receive text messages and calls. |  |  |  |  |  |  |  |
| 1. I would feel anxious because I could not stay in touch with my family and friends. |  |  |  |  |  |  |  |
| 1. I would be nervous because I could not know if someone had tried to contact me. |  |  |  |  |  |  |  |
| 1. I would feel anxious because my continuous contact with my family and friends would be broken. |  |  |  |  |  |  |  |
| 1. I would be nervous because I would be disconnected from my online identity. |  |  |  |  |  |  |  |
| 1. I would be uncomfortable because I could not keep up with social media and online networks. |  |  |  |  |  |  |  |
| 1. I would feel awkward because I could not check my notifications of updates from my contacts and online networks. |  |  |  |  |  |  |  |
| 1. I would feel anxious because I could not check my email. |  |  |  |  |  |  |  |
| 1. I would feel strange because I would not know what to do |  |  |  |  |  |  |  |
